# Magnolol Administration in Normotensive Young Spontaneously Hypertensive Rats Postpones the Development of Hypertension: Role of Increased PPAR Gamma, Reduced TRB3 and Resultant Alleviative Vascular Insulin Resistance

**DOI:** 10.1371/journal.pone.0120366

**Published:** 2015-03-20

**Authors:** Xiangyan Liang, Wenjuan Xing, Jinxiao He, Feng Fu, Wei Zhang, Feifei Su, Fange Liu, Lele Ji, Feng Gao, Hui Su, Xin Sun, Haifeng Zhang

**Affiliations:** 1 Experiment Teaching Center, Fourth Military Medical University, Xi'an, China; 2 Department of Physiology, Fourth Military Medical University, Xi'an, China; 3 Department of Pediatrics, Xijing Hospital, Fourth Military Medical University, Xi'an, China; 4 Department of Cardiology, Tangdu Hospital, Fourth Military Medical University, Xi'an, China; 5 Department of Geratology, Xijing Hospital, Fourth Military Medical University, Xi’an, China; University of Utah School of Medicine, UNITED STATES

## Abstract

Patients with prehypertension are more likely to progress to manifest hypertension than those with optimal or normal blood pressure. However, the mechanisms underlying the development from prehypertension to hypertension still remain largely elusive and the drugs for antihypertensive treatment in prehypertension are absent. Here we determined the effects of magnolol (MAG) on blood pressure and aortic vasodilatation to insulin, and investigated the underlying mechanisms. Four-week-old male spontaneous hypertensive rats (SHR) and age-matched normotensive Wistar-Kyoto (WKY) control rats were used. Our results shown that treatment of young SHRs with MAG (100 mg/kg/day, o.g.) for 3 weeks decreased blood pressure, improved insulin-induced aorta vasodilation, restored Akt and eNOS activation stimulated by insulin, and increased PPARγ and decreased TRB3 expressions. In cultured human umbilical vein endothelial cells (HUVECs), MAG incubation increased PPARγ, decreased TRB3 expressions, and restored insulin-induced phosphorylated Akt and eNOS levels and NO production, which was blocked by both PPARγ antagonist and siRNA targeting PPARγ. Improved insulin signaling in HUVECs by MAG was abolished by upregulating TRB3 expression. In conclusion, treatment of young SHRs with MAG beginning at the prehypertensive stage decreases blood pressure via improving vascular insulin resistance that is at least partly attributable to upregulated PPARγ, downregulated TRB3 and consequently increased Akt and eNOS activations in blood vessels in SHRs.

## Introduction

Prehypertension, which is blood pressure readings with a systolic pressure from 120 to 139 mm Hg or a diastolic pressure from 80 to 89 mm Hg, is an American medical classification for cases where a person's blood pressure is elevated above normal, but not to the level considered hypertension (high blood pressure) [1]. A statistical analysis of disease-free adult NHANES (national health and nutrition examination surveys) participants was conducted from 1999 to 2006. Overall prevalence of prehypertension in disease-free adults was 36.3% [2]. There is now good clinical evidence that patients with high-normal blood pressure (prehypertension) are more likely to progress to manifest hypertension than patients with optimal or normal blood pressure [3,4]. Even some people with prehypertension who do not progress to hypertension may be at increased risks of cardiovascular disease (CVD) mortality, especially with stroke mortality [5]. Treatment of prehypertension with candesartan appeared to be well tolerated and reduced the risk of incident hypertension during the study period [3]. Thus, treatment of prehypertension appears to be feasible. Our latest results showed that long-term exercise beginning at the prehypertensive stage may help to limit the progression of hypertension [6]. However, the mechanisms underlying the development from prehypertension to hypertension still remain largely elusive and the drugs for antihypertensive treatment in prehypertension are absent. Magnolol (MAG) is a biologically active compound of Cortex magnolia officinalis, a Chinese medicinal herb. MAG is commonly used as a blood-quickening and stasis-dispelling agent in traditional Chinese medicine. Effects of MAG in the cardiovascular system include prevention of myocardial ischemic-reperfusion injury [7,8] and anti-atherosclerosis [9–15]. However, its effect on blood pressure, especially in prehypertension remains unknown.

In our previous studies, young spontaneously hypertensive rats (ySHRs) with normal blood pressure exhibited significantly decreased aortic vasodilatation to insulin [16,17], and exercise mitigates hypertension through improving vascular insulin sensitivity of resistance vessels [6]. There is evidence that MAG might have beneficial effects on glucose metabolism by activating the insulin signaling pathway [18–20]. But whether and how MAG influences blood pressure and insulin-induced vasodilation of resistance vessels in normotensive ySHRs remain elusive. In addition, tribbles 3 (TRB3) affects insulin signaling and action by inhibiting Akt phosphorylation [21], but its upstream signaling remains unknown. Activation of peroxisome proliferator-activated receptor-gamma (PPARγ) may have direct effects on important genes involved in insulin signaling [22,23]. Experimental evidence showed that compared with age-matched WKY rats, the expression levels of PPARγ in vascular tissues were significantly decreased, but systolic blood pressure were significantly increased [24]. It is reported that MAG actives PPARγ in vitro [25]. However, whether MAG affects PPARγ and TRB3 expressions remains unclear.

Therefore, the aims of this study were to (1) determine effects of MAG on blood pressure and aortic vasodilatation to insulin; (2) investigate the underlying mechanism with a special focus on PPARγ and TRB3 expressions and vascular insulin signaling.

## Materials and Methods

### Materials

Unless otherwise indicated, all reagents were obtained from Sigma (St. Louis, MO). The antibodies of PPARγ, TRB3, Akt, pAkt, eNOS and peNOS were purchased from Cell Signaling Technology (Danvers, MA). Chariot was purchased from Active Motif (Carlsbad, CA). MAG was obtained from Shaanxi xuhuang Plant Technology Development Co., Ltd. (Xi’an, China). For in vivo treatment, MAG was diluted in distilled water, for in vitro treatment MAG was diluted in DMSO. Stock solutions of Phenylephrine (PE), acetylcholine (ACh), S-nitroso-N-acetylpenicillamine (SNAP), insulin, and N^ω^-nitro-L-argininemethyl ester (L-NAME) were prepared with distilled water.

### Ethics Statement

The experiments were performed in adherence with the National Institutes of Health Guidelines for the Use of Laboratory Animals. All experiments involving rats were reviewed and approved by the Ethics Committee for animal care and use of Fourth Military Medical University, P.R. China. The use of human umbilical vein endothelial cell lines (HUVEC) was reviewed and approved by Ethics Committee of Xijing Hospital, Fourth Military Medical University, P.R. China, and written informed consent was given by persons donating umbilical cords for use of this sample in research as described in our previous study[6,16].

### Experimental protocols

All procedures in animals were performed in accordance with Guidelines for the Use of Laboratory Animals and were approved by the local authorities for animal research. Four-week-old male spontaneous hypertensive rats (SHRs) and age-matched normotensive Wistar-Kyoto (WKY) control rats were purchased from Vital River Laboratories (Beijing, China). SHRs were randomized into three groups and treated daily for 3 weeks by gavage with vehicle alone (distilled water), MAG (100 mg/kg/day), or MAG (100 mg/kg/day) + bisphenol A diglycidyl ether (BADGE, a PPARγ antagonist, 30 mg/kg/day). WKY rats were randomized into two groups and treated daily by gavage with distilled water or MAG (100 mg/kg/day). Systolic blood pressure (SBP) was measured noninvasively using a tail cuff according to standard procedures described previously [6,16]. Reported systolic blood pressure values are the average of three sequential blood pressure measurements that were within 10 mmHg of each other. After 3 weeks of treatment, the animals were anesthetized with the 20% urethane. Blood samples were obtained by puncture from vena cava and immediately centrifuged to determine fasting blood glucose levels. Aortae from the heart to the iliac bifurcation were removed from rats and mounted in ice cold Krebs buffer and continuously gassed with a mixture of 95% O_2_–5% CO_2_ (pH 7.4).

### Organ chamber experiments

Organ chamber experiments were performed as previously described [17,26]. Briefly, vessels were cut into 3-mm rings and suspended in organ chambers containing 10 mL of Krebs solution and aerated with 95% O_2_–5% CO_2_ at 37°C. Krebs buffer consisting of (in mM): NaCl 118, KCl 4.8, CaCl_2_·2H_2_O 2.5, MgCl_2_·6H_2_O 2.5, NaH_2_PO_4_·2H_2_O 1.2, NaHCO_3_ 8.5, and Glucose·H_2_O 11.0. In some segments, the endothelium was mechanically removed by pulling silk suture through the vessel. Rings were connected with a force transducer to record changes via a BL-420 data acquisition system (Chengdu Taimeng). The vessel segments were gradually stretched over 60 min to a baseline tension of 1 g, which was perpetuated throughout the procedure, and were allowed to equilibrate for another 30 min. Then, the rings were pre-contracted with PE (10^−6^ mol/L). Once a stable contraction was achieved, dose-response curves measuring vasodilation in response to insulin were obtained by adding increasing concentrations of insulin (10^−10^~10^−6^ mol/L) to the perfusate. To assess the influence of nitric oxide (NO, a vasodilator) on insulin-induced vasorelaxation, part of the rings was pre-treated with Nω- nitro-L-arginine methyl ester (L-NAME, 0.5 mmol/L), an inhibitor of NO synthase (NOS), 30 min before insulin stimulation. After vasorelaxation was assessed, substances were washed out thoroughly and the aortic segments were subjected to immunoblotting measurement.

### Glucose and insulin determinations

Fasting blood glucose was determined with a blood glucose meter (Lifescan). Insulin levels were measured with the RIA test kit (Peninsula Laboratories, Belmont, CA) [17].

### Immunoblotting

Proteins were separated on SDS-PAGE gels, transferred to PVDF (polyvinylidenedifluoride, Millipore), the membranes were blocked with 5% BSA and incubated overnight at 4°C with antibodies directed against PPARγ, TRB3, Akt, pAkt, eNOS or peNOS. After washing blots to remove excessive primary antibody binding, blots were incubated for 1 h with secondary antibody. Antibody binding was detected via enhanced chemiluminescence (Millipore). Film was scanned with ChemiDocXRS (Bio-Rad Laboratory, Hercules, CA). Immunoblot band intensity was analyzed with Lab Image software.

### Cell culture, Small interfering RNA transfection and chariot-mediated antibody delivery

HUVECs were cultured in endothelial cell basal medium containing 2% fetal bovine serum and endothelial growth supplements. Experiments were performed with cells of the passages 5~8 when grown to 70~80% confluence. This study was approved by our institutional review board. For experiments evaluating MAG treatment, cells were serum-starved overnight and then incubated for 30 min in the absence or presence of inhibitors BADGE (50 μmol/L) prior to MAG treatment.

Small interfering RNA (siRNA) specifically targeting PPARγ mRNA was purchased from Santa Cruz Biotechnology. Twenty-four hours prior to transfection, cells were plated onto a 6-well plate or a 96-well plate at 50–70% confluency. HUVECs were then transfected with PPARγ siRNA or scrambled control by Lipofectamine 2000 (Invitrogen, Carlsbad, CA) according to the manufacturer’s instructions [16]. At 48 hours after transfection, cells were serum-starved for 6 hours and treated with MAG (10 μmol/L). To determine the insulin-induced signaling pathway, after incubation of MAG for 48 hours, the medium was changed into ordinary culture medium and then the HUVECs were exposed to 10^−7^ mol/L insulin for 30 min. The efficiency of siRNA transfection was detected by Western blot analysis at 48 hour after siRNA transfection.

To increase TRB3 expression, a macromolecular protein delivery system, Chariot (Active Motif, Carlsbad, CA), was used following the manufacturer’s instructions as described by Taguchi et al [27]. Briefly, protein was first incubated with the protein delivery agent Chariot (1:1 vol/vol) at room temperature for 30 min to allow the complex to form. HUVECs were transferred to a sterile 24-well cell culture plate, overlaid with 200 μl of Chariot/protein complex, and mixed gently. DMEM (300 μl) was added, and HUVECs were incubated for 1 hour at 37°C. Additional DMEM (500 μl) containing 10% fetal bovine serum was then added, and HUVECs were further incubated for 2 hours at 37°C. After that, HUVECs were subjected to the further measurements. The efficiencies of protein delivery were detected by Western blot analysis.

### Quantification of NO release in endothelial cells

Total NO production in culture medium or by aortic segments was determined by measuring the concentration of nitrite, a stable metabolite of NO, with a modified Griess reaction method as reported previously [28].

### Statistical analysis

Data in the figures are presented as means ± SEM and were analyzed by Student’s t-test between two groups or by ANOVA when three or more groups were compared. In all statistical comparisons, a *P* value < 0.05 or less were considered statistically significant.

## Results

### Effects of MAG on blood pressure and physiological parameters in WKY and SHRs

**Fig 1:**
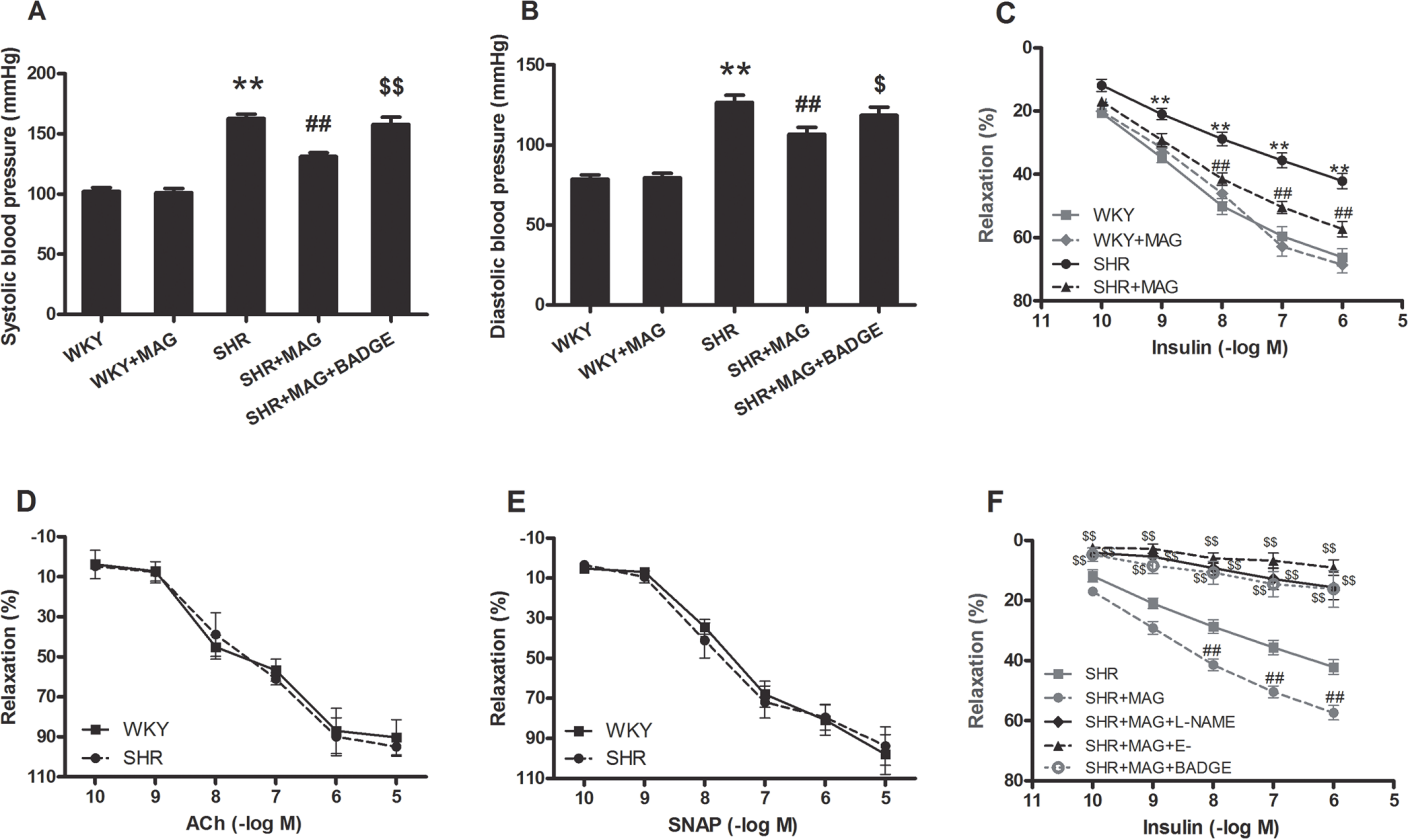
The effects of magnolol on blood pressure and insulin-induced vasodilatation in rats. (A) The effect of magnolol of systolic blood pressure (SBP). (B) The effect of magnolol on diastolic pressure (DBP). Impairment of insulin-induced vasodilatation and the effect of magnolol on insulin-induced vasodilatation in aortic segments from SHRs (C). Either ACh (D) or SNAP (E) elicited a significant vasorelaxation effects in aortic vessels from both WKY rats and SHRs, and there was no difference in aortic vasorelaxation between the two strains in response to both vasodilators respectively. (F) Insulin-induced relaxation was abolished either by removal of the endothelium or by pretreatment with L-NAME of the vessels from SHRs by MAG treatment, and also was inhibited by PPARγ antagonist bisphenol A diglycidyl ether (BADGE) pretreatment in vivo. Mag, magnolol; L-NAME, N^ω^-nitro-L-argininemethyl ester; E^−^, endothelium denudation. n = 6, **P<0.01 vs. WKY; ^##^ *P*<0.01 vs. SHR; ^$$^ *P*<0.01 vs. SHR+MAG.

Blood pressure of WKY and SHRs before MAG administration were measured. The systolic blood pressure (SBP) and diastolic blood pressure (DBP) of WKY was 104 ± 8.4 mmHg and 80 ± 6.6 mmHg, respectively; SBP and DBP of SHRs was 112 ± 7.6 mmHg and 87 ± 6.4 mmHg, respectively. There was no difference between WKY and SHRs. After MAG administration 3 weeks, compared with WKY rats, the body weight, fasting glucose (9.3 ± 0.3 mmol/L vs. 5.6 ± 0.4 mmol/L, n = 8, *P*<0.05) and fasting insulin levels (22.0 ± 0.3 μU/ml vs. 18.4 ± 0.5 mmol/L, *P*<0.05), systolic blood pressure (SBP) and diastolic blood pressure (DBP) of SHRs were significantly increased (Fig. 1A and 1B). MAG therapy significantly reduced fasting glucose (6.6 ± 0.2 mmol/L, *P*<0.05), fasting insulin levels (18.9 ± 1.3μU/ml, *P*<0.05), SBP and DBP, but did not alter body weight in SHRs after 3 weeks of administration compared with vehicle treatment.

### Effects of MAG on insulin-induced vasorelaxation of the aortic segments

To evaluate the effects of MAG on vascular response to insulin, we isolated aortic rings from rats and investigated the relaxation of aortic segments in response to insulin. As shown in Fig. 1C, the ability of insulin to cause dose-dependent vasorelaxation was significantly impaired in vascular segments from SHRs when compared with samples from WKY rats, indicating that vasodilator actions in response to insulin of SHRs have been impaired. From Fig. 1D and 1E we found that both ACh and SNAP elicited significant vasorelaxation effects in aortic vessels from WKY rats and SHRs, but vasorelaxation between the two strains in response to the ACh or SNAP was not different, indicating that the impaired insulin signaling, but not the capacity of endothelium in releasing NO or of smooth muscle in relaxation from SHRs, is responsible for the blunted vasorelaxation in response to insulin in SHRs. Importantly, 3 weeks of MAG treatment increased vasodilator response to insulin, but the PPARγ antagonist BAGDE inhibited this effect of MAG. In addition, insulin-induced relaxation was abolished either by removal of the endothelium or by pretreatment with L-NAME of the vessels from SHRs by MAG treatment (Fig. 1F).

### Effects of MAG on vascular PPARγ and TRB3 expressions and insulin-stimulated vascular Akt/eNOS signaling

**Fig 2:**
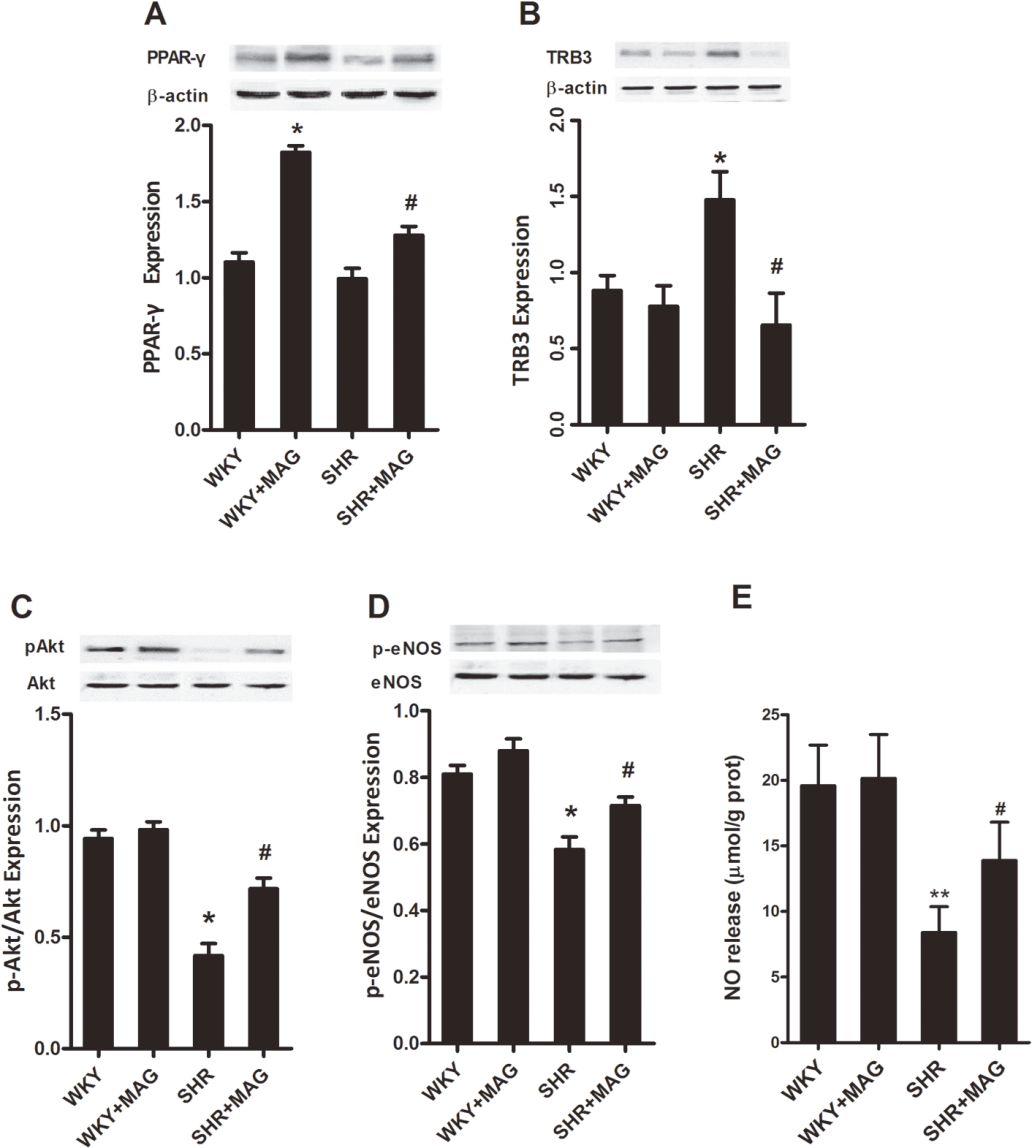
A and B, Representative blots and quantitative densitometry of PPARγ and TRB3 in the aortic segments from control or magnolol-treated WKY and SHRs. C and D, Representative blots of total Akt and phosphorylated Akt and the ratio of phosphorylated Akt/total Akt, total eNOS and phosphorylated eNOS and the ratio of phosphorylated eNOS/total eNOS in the aortic segments with insulin treatment from control or magnolol-treated WKY and SHRs, and NO release (E) in the rat aortas. Data obtained from quantitative densitometry were presented as mean ± SEM of at least 5 independent experiments. M, magnolol. *P<0.05 vs. WKY, ^#^ *P*<0.05 vs. SHRs.

To determine the molecular mechanisms by which MAG treatment increased vasodilator response to insulin in SHRs, we examined the expression levels of PPARγ and TRB3 in vascular tissues from SHRs by immunoblotting. As shown in Fig. 2A and 2B, the expression levels of PPARγ in SHR aortae were similar to those in age-matched WKY rats, but TRB3 levels were significantly increased. MAG treatment significantly increased the expressions of PPARγ, and decreased the TRB3 levels in SHRs (*P*<0.05). These results firstly suggest that MAG may act as a PPARγ agonist and upregulate its levels *in vivo*.

To investigate the effect of MAG in insulin-stimulated vascular Akt/eNOS signaling, we examined the expression of Akt, eNOS, and phosphorylated Akt and eNOS levels in vascular tissues from SHRs by immunoblotting. As illustrated in Fig. 2C, 2D and 2E, the levels of phosphorylated Akt and eNOS and NO release were dramatically decreased in vascular tissue of SHRs (*P*<0.05) in comparison to those of age-matched WKY rats. Treatment with MAG for 3 weeks resulted in increased aortic phosphorylated Akt and eNOS and NO release in SHRs (*P*<0.05). These results indicate that MAG treatment is able to improve insulin signaling transduction possibly trough increasing PPARγ and decreasing TRB3 levels in SHRs.

### Effects of MAG incubation on PPARγ, TRB3 expressions and insulin-stimulated Akt/eNOS/NO signaling in cultured human umbilical vein endothelial cells

**Fig 3:**
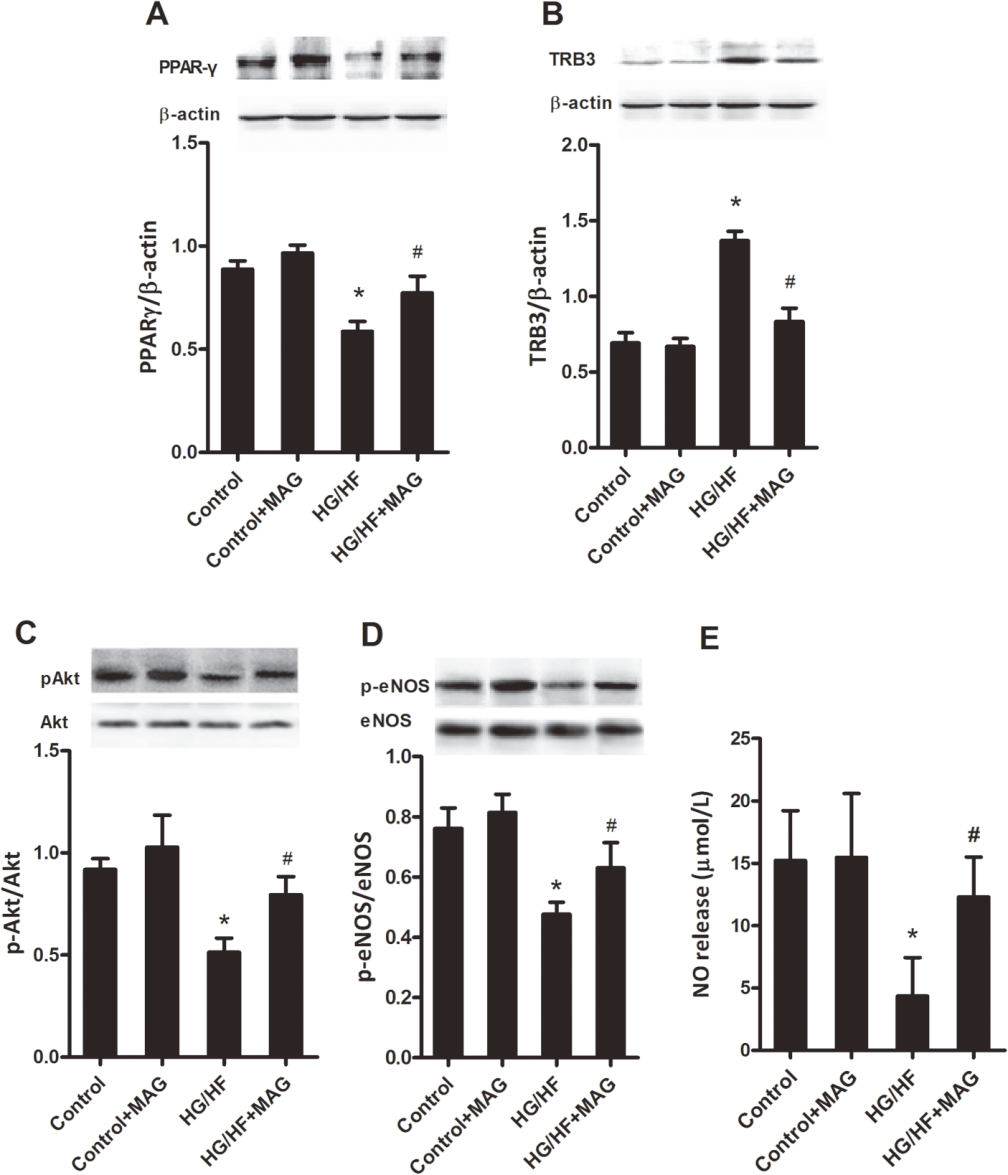
Effects of magnolol incubation on PPARγ expression and insulin-stimulated Akt/endothelial NO synthase (eNOS)/NO signaling in human umbilical vein endothelial cells. Representative western blots showing PPARγ (A), TRB3 (B), phosphorylated Akt / Akt (C), phosphorylated eNOS / eNOS (D), and NO release (E) in the conditioned medium. HG/HF, high glucose/high fat treatment. All values are presented as mean ± SEM. n = 5–6. *P<0.05 vs. Control, ^#^ *P*<0.05 vs. HG/HF.

To directly investigate whether MAG increases PPARγ, decreases TRB3 expressions and improve insulin signaling transduction in endothelial cells in vitro, we cultured HUVECs in media containing glucose (25 mmol/L) and saturated FFA palmitate (16:0; 500 μmol/L), a common fatty acid (HG/HF) [29]. As shown in Fig. 3, after 18 h of HG/HF incubation, PPARγ expression was significantly decreased, TRB3 expression was significantly increased, the phosphorylated Akt and eNOS were dramatically decreased and NO release was reduced. But MAG reversed these tendency and finally increased NO release (*P*<0.05). These data suggest that MAG upregulates PPARγ, downregulates TRB3 and reduces insulin sensitivity obtuseness in the presence of HG/HF.

### PPARγ antagonist decreased effects of MAG incubation on PPARγ expressions and insulin-stimulated signaling pathway in human umbilical vein endothelial cells

**Fig 4:**
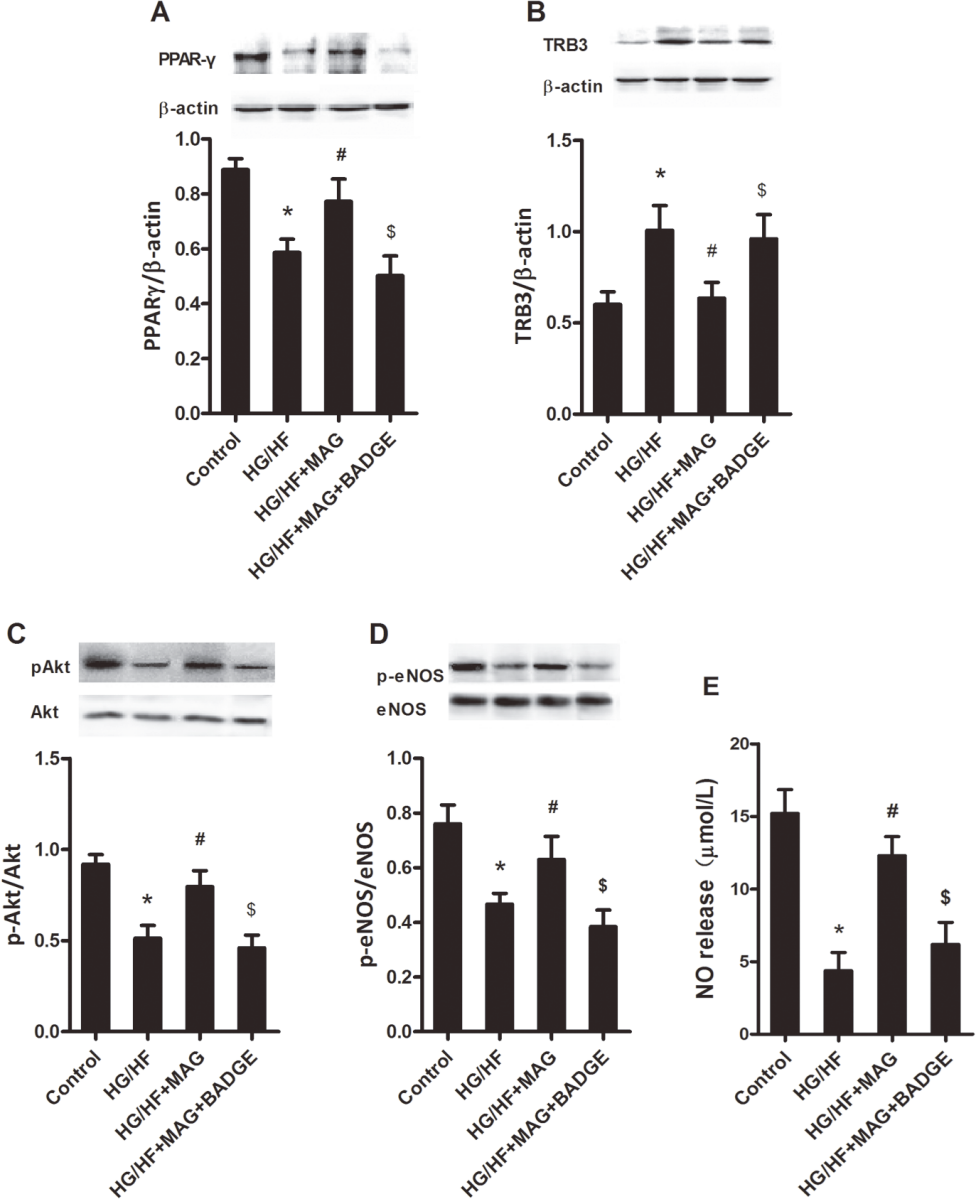
PPARγ antagonist blocked effects of MAG incubation on PPARγ expression and insulin-stimulated signaling pathway in human umbilical vein endothelial cells. Representative western blots showing PPARγ (A), TRB3 (B), phosphorylated Akt / Akt (C), and phosphorylated eNOS / eNOS (D), and NO release (E) in the conditioned medium. HG/HF, high glucose/high fat treatment; Mag, magnolol; BADGE, PPARγ antagonist bisphenol A diglycidyl ether (BADGE). All values are presented as mean ± SEM. n = 5–6. *P<0.05 vs. Control, ^#^ *P*<0.05 vs. HG/HF, ^$^ *P*<0.05 vs. HG/HF+BADGE.

To further investigate the role of MAG in increasing PPARγ expression and activation, decreasing TRB3 expression accordingly and increasing insulin sensitivity, we used BADGE, a specifical PPARγ antagonist. As shown in Fig. 4, BADGE pretreatment significantly antagonized the effects of MAG on PPARγ and TRB3 expressions, and phosphorylated Akt, eNOS and NO levels, although BADGE itself has no effect on these proteins (data not shown). Thus the downregulation of TRB3 and sensibilization of insulin by MAG were dependent on PPARγ upregulation and activation in HUVECs.

### PPARγ inhibition decreased effects of MAG incubation on PPARγ expressions and insulin-stimulated signaling pathway in human umbilical vein endothelial cells

**Fig 5:**
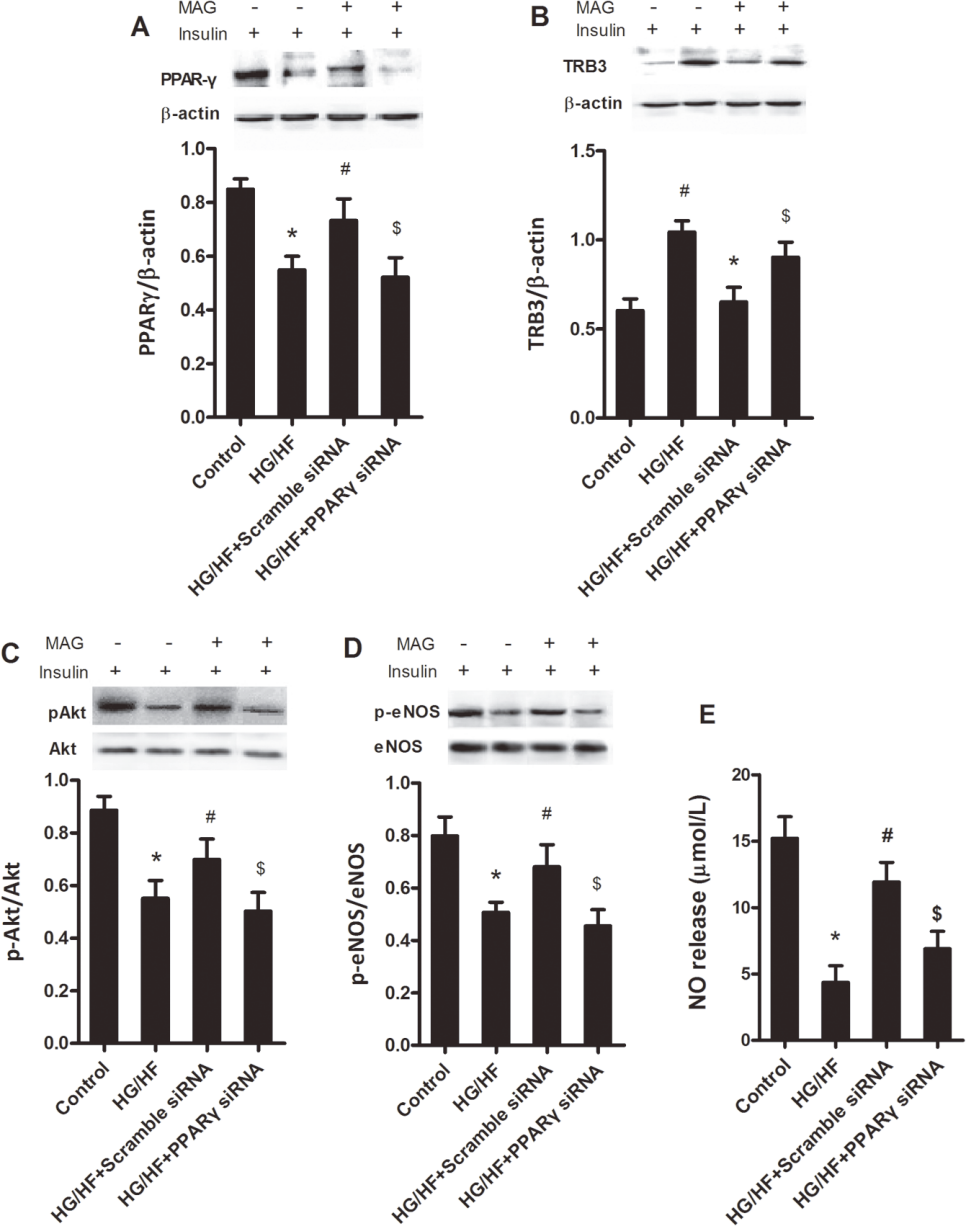
PPARγ inhibition decreased effects of MAG incubation on PPARγ expression and insulin-stimulated signaling pathway in human umbilical vein endothelial cells. Representative western blots showing PPARγ (A), TRB3 (B), phosphorylated Akt / Akt (C), and phosphorylated eNOS / eNOS (D), and NO release (E) in the conditioned medium. HG/HF, high glucose/high fat treatment; Mag, magnolol. All values are presented as mean ± SEM. n = 5–6. *P<0.05 vs. Control, ^#^ *P*<0.05 vs. HG/HF, ^$^ *P*<0.05 vs. HG/HF+Scrmbled siRNA.

To directly investigate whether MAG enhances insulin sensitivity through PPARγ, we used siRNA targeting PPARγ to suppress PPARγ expression in HUVECs. PPARγ expression was significantly decreased by siRNA of PPARγ, whereas its endogenous expression was not changed by scrambled siRNA sequence (Data not shown). PPARγ siRNA transfection significantly suppressed increasing PPARγ expressions in HUVECs incubated by MAG, but scrambled siRNA transfection did not changed PPARγ expressions (Fig. 5A). In addition, suppressed PPARγ expression by siRNA significantly blocked MAG incubation-reduced TRB3 expression (Fig. 5B) and -increased Akt/eNOS activations and NO release by insulin in HUVECs (Fig. 5C, 5D and 5E).

### Improved insulin signaling in human umbilical vein endothelial cells by MAG were abolished by upregulating TRB3 expression

**Fig 6:**
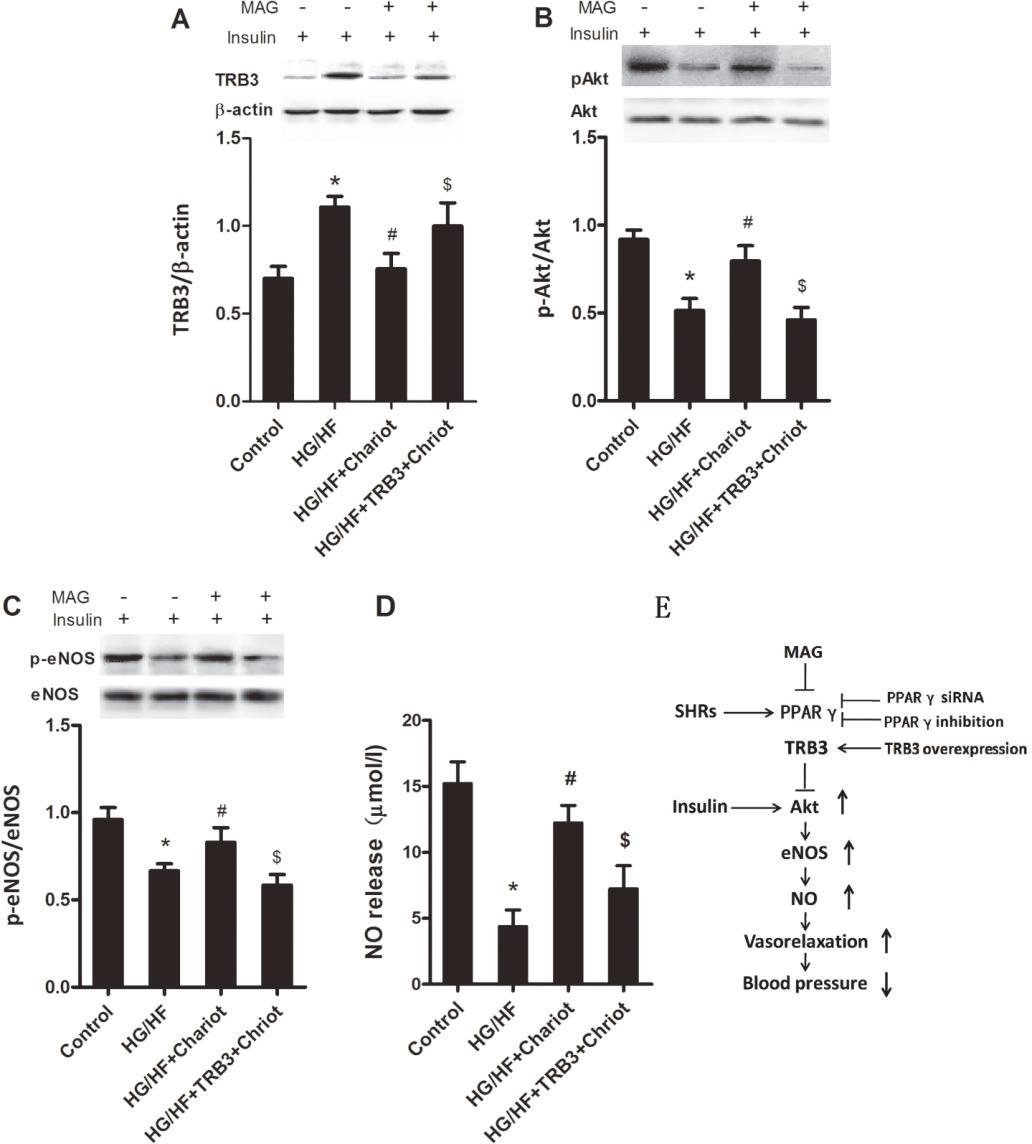
Improved insulin signaling of human umbilical vein endothelial cells by magnolol were abolished by upregulating TRB3 expression. Representative western blots showing TRB3 (A), phosphorylated Akt / Akt (B), and phosphorylated eNOS / eNOS (C). Mag, magnolol, (D) NO release in the conditioned medium. (E) Proposed mechanisms of magnolol attenuates the vascular insulin resistance and reduces blood pressure. All values are presented as mean ± SEM. n = 5–6. *P<0.05 vs. Control, ^#^ *P*<0.05 vs. HG/HF, ^$^ *P*<0.05 vs. HG/HF+Chariot.

To provide further evidence for a relationship between TRB3 and MAG induced-improvement of insulin sensitivity in HUVECs, we used Chariot for delivering TRB3 to cultured HUVECs. As shown in Fig. 6A, the reduction of TRB3 expression in cultured HUVECs by MAG was reversed by delivery of TRB3 to HUVECs. Insulin-induced activation of Akt and eNOS and NO release were also significantly reduced (Fig. 6B and 6C). Thus TRB3 overexpression abolished the effects of MAG on insulin sensitivity in HUVECs.

## Discussion

The present study is the first report demonstrating that MAG administration in prehypertension delays blood pressure increasing in SHRs by improving vascular insulin sensitivity of blood vessels. More importantly, we demonstrated that MAG attenuates vascular insulin resistance by upregulating PPARγ, downregulating TRB3 expressions and consequently increasing Akt and eNOS activation in SHRs, which is causally linked to the ability of MAG to enhance the vasodilator actions of insulin that help to decrease blood pressure in prehypertension.

Magnolol (MAG) is a biologically active compound of Cortex magnolia officinalis, a Chinese medicinal herb commonly used as a blood-quickening and stasis-dispelling agent. Its chemical name is 5,5’-diallyl-2,2’-dihydroxy diphenyl. Effects of MAG in the cardiovascular system has been investigated [7–15], however, its effects on blood pressure remain unknown. Our present study showed that 3 weeks treatment of SHRs with MAG beginning at prehypertensive stage significantly reduced SBP and DBP when compared with SHRs treated with vehicle, indicating that MAG therapy in the prehypertensive stage may attenuate the progression from prehypertension to hypertension. So MAG may be beneficial for the patients with prehypertension, especially for hypertension-prone individuals. It's worth noting that we didn’t know the effects of MAG on established hypertension. Nevertheless, the effects of long-term oral administration (7 weeks) of honokiol on hypertension in adult SHRs had been investigated previously, which is the isomer of MAG and the phenolic compound purified from the medical plant Cortex magnolia officinalis. It was reported that honokiol administration significantly decreased systolic blood pressure after hypertension developed steadily (Zhang et al. Antihypertensive potential and mechanism of effects of honokiol on SHRs (Doctoral thesis). Guangzhou University of Chinese Medicine. 2009.). Whether MAG acts like its isomer honokiol needs to be investigated in the future.

One approach for assessing vascular function is to measure endothelium-dependent vasorelaxation of arteries in response to ACh and insulin. Indeed, in this study vasorelaxation in response to ACh in 7-wk-old SHRs is normal while the response to insulin is impaired. ACh is a classic cholinergic agonist that activates eNOS by a calcium-dependent mechanism. Unchanged vasodilator response to ACh may be attributable to the unchanged [Ca^2+^]_i_ and related NO release [30]. Vascular insulin resistance has been demonstrated existing in prehypertension, which may be a cause of hypertension development [16,17]. Others’ and our previous studies have reported that vascular insulin resistance, which is characterized by blunted insulin-induced vasorelaxation or imbalance of vascular insulin signaling, occurs before systemic insulin resistance in hypertensive and high-fat diet models [17,31]. The isolated endothelial dysfunction with respect to insulin that we observed may be an early feature of vasomotor abnormalities characteristic of the hypertensive process that precedes a generalized endothelial dysfunction (with abnormal response to ACh, which is usually present after the age of 12 weeks in SHRs) found in well-advanced essential hypertension. Importantly, long-term exercise alleviates hypertension via improving vascular insulin sensitivity in SHRs [6]. Here we proved that 3 weeks of MAG treatment markedly improved vascular insulin sensitivity as evidenced by increased vasodilator response to insulin. Accordingly, these findings suggest that MAG decreases blood pressure through improving vascular insulin sensitivity of blood vessels in prehypertension.

It is reported that insulin has vasodilator actions by Akt and eNOS-mediated pathway [32]. In stroke-prone SHRs, insulin-like growth factor-1 receptor and its downstream signaling, such as phosphorylated Akt was downregulated [33]. Our previous and present studies also demonstrated that young prehypertensive SHRs have blunted insulin-induced vasorelaxation and impaired insulin signaling (PI3K/Akt/eNOS) and decreased NO production in the vasculature [6,16,17]. Thus, these suggest that blunted insulin-induced vasorelaxation in SHRs is accompanied by defects in Akt/eNOS mediated pathways in endothelium. Our present data showed that treatment with MAG resulted in increased aortic phosphorylated Akt and eNOS levels in SHRs. In HUVECs, MAG reduced insulin sensitivity impairment in the presence of HG/HF by increasing phosphorylated Akt and eNOS, and finally increased NO release. Taken together, these results suggest that MAG treatment at prehypertensive stage may restore impaired insulin signaling pathways, increase vascular insulin sensitization and subsequent decrease systemic blood pressure.

As a ligand-activated transcription factor, PPARs regulate transcription of target genes by heterodimerizing with the retinoid X receptor and binding to PPAR response elements of regulatory promoter regions of target genes. PPARγ is not only expressed in adipose tissue but also expressed in cardiovascular system cells, such as cardiac myocytes, endothelial cells and vascular smooth muscle cells. It is reported that compared with age-matched WKY rats, the expression levels of PPARγ in vascular tissues of SHRs were significantly decreased, but systolic blood pressure were increased [24]. Activation of PPARγ may have direct effects on important genes involved in insulin signaling [21,23]. It is reported that MAG activates PPARγ in vitro [25]. Our present study demonstrated that treatment with MAG for 3 weeks resulted in increased PPARγ expressions and insulin signaling in SHRs. In the presence of HG/HF in HUVECs, MAG incubation increased the expressions of PPARγ and insulin signaling. Importantly, both PPARγ antagonist and siRNA targeting PPARγ blocked the effect of MAG on PPARγ expressions and insulin signaling. Therefore, MAG treatment may restore the impaired insulin signaling, that is, insulin-induced Akt and eNOS phosphorylations, through increasing and acting PPARγ in young SHRs vascular tissues.

TRB3, a mammalian tribbles homologue, whose chromosomal region 20p13-p12 has been linked to human type 2 diabetes, affects insulin signaling and action by inhibiting Akt phosphorylation [21], which was also observed in the present study. The overexpression of TRB3 inhibited Akt phosphorylation induced by insulin, but decreased TRB3 expressions by antisense oligonucleotide technology, Akt phosphorylation induced by insulin significantly increased [34]. The prevalent TRB3 missense Q84R polymorphism is significantly associated with several insulin resistance [35]. These results indicated that TRB3 impairs insulin signaling through inhibiting Akt phosphorylation, but its upstream mechanisms remains unknown. Interestingly, our results showed that MAG treatment not only significantly increased the expressions of PPARγ in SHRs, but also in WKY, which was consistent with our previous study [17]. However, MAG treatment only significantly decreased the expressions of TRB3 in SHRs, but not in WKY. The main reason may be that MAG just regulates the expressions of TRB3 via upregulating the transcription factor PPARγ in pathological conditions like hypertension, but do not act in normal conditions in which there is just a small quantity of TRB3. Additionally, we found that, both PPARγ antagonist and siRNA targeting PPARγ blocked the effects of MAG on decreasing TRB3 expressions. So MAG, a PPARγ agonist, as evidenced by increased PPARγ expressions and activation, inhibits its downstream TRB3 expressions, enhances Akt and eNOS activations, and finally decreases blood pressure.

In summary, our findings demonstrate for the first time that treatment of young SHRs with MAG at the prehypertensive stage decreases blood pressure via improving vascular insulin resistance. It may be attributable to upregulated PPARγ, downregulated TRB3 and consequently increased Akt and eNOS activations in blood vessels in SHRs (Fig. 6D). These results indicate that MAG may be used as an antihypertensive drug early used at the prehypertensive stage.
